# Effects of feeding untreated, pasteurized and acidified waste milk and bunk tank milk on the performance, serum metabolic profiles, immunity, and intestinal development in Holstein calves

**DOI:** 10.1186/s40104-017-0182-4

**Published:** 2017-06-01

**Authors:** Yang Zou, Yajing Wang, Youfei Deng, Zhijun Cao, Shengli Li, Jiufeng Wang

**Affiliations:** 1 State Key Laboratory of Animal Nutrition, Beijing Engineering Technology Research Center of Raw Milk Quality and Safety Control, College of Animal Science and Technology, China Agricultural University, Beijing, 100193 China; 2 Beijing Dairy Cattle Center, Beijing, 100192 China; 3 College of Veterinary Medicine, China Agricultural University, Beijing, 100193 China

**Keywords:** Acidified waste milk, Calf, Intestinal development, Pasteurized waste milk, Serum metabolism, Waste milk

## Abstract

**Background:**

The present experiment was performed to assess the effects of different sources of milk on the growth performance, serum metabolism, immunity, and intestinal development of calves. Eighty-four Holstein male neonatal calves were assigned to one of the following four treatment groups: those that received bunk tank milk (BTM), untreated waste milk (UWM), pasteurized waste milk (PWM), and acidified waste milk (AWM) for 21 d.

**Results:**

Calves in the BTM and AWM groups consumed more starter (*P* < 0.05) than those in the UWM group. Average daily gain in the UWM group was the highest (*P* < 0.05). Calves exhibited the highest (*P* < 0.05) serum total protein, albumin, total cholesterol, high density lipoprotein, triglycerides, growth hormone, immunoglobulin (Ig) A and IgM concentrations in the UWM group, highest malondialdehyde and tumor necrosis factor-α in the PWM group (*P* < 0.05), and highest glutathione peroxidase and IgG in the BTM group (*P* < 0.05). The jejunum and ileum of the calves in all treatments presented a slight inflammatory response. The jejunal inflammation scores were higher (*P* < 0.05) in the UWM and AWM groups than the BTM group; the ileal inflammation scores increased more (*P* < 0.05) in the AWM group than the BTM group. Jejunal immunohistochemical scores (IHS) were higher (*P* < 0.05) in the PWM and AWM groups than the BTM group. Compared to the other three groups, calves feeding on BTM had lower (*P* < 0.05) ileal IHS. Jejunal *interleukin(IL)-1β*, *IL-8*, and *IL-10* mRNA expression in the UWM group was the highest (*P* < 0.05). Calves fed AWM increased (*P* < 0.05) mRNA expression of *IL-8* and *toll like receptor 4 (TLR-4)* in the jejunum and *IL-8*, *IL-1β*, *IL-6*, *IL-8*, and *IL-10* in the mesenteric lymph nodes.

**Conclusions:**

Overall, bunk tank milk is the best choice for calf raising compared to waste milk. The efficiency of feeding pasteurized and acidified waste milk are comparable, and the acidification of waste milk is an acceptable labor-saving and diarrhea-preventing feed for young calves.

## Background

All non-saleable milk on farms, including colostrum, transitional milk, high somatic cell content milk, and milk from cows treated with veterinary drugs due to diseases, is classified as waste milk (WM) [1]. The use of WM in calf feeding is considered economical but controversial [2, 3]. The major concern in using WM is the excessive amounts of bacteria and antibiotics [1]. The pasteurization of WM has been suggested to minimize the occurrence of pathogens such as *Salmonella* [4–6]. Additionally, different sterilization method of WM may result in varition of growth performance and development of calves. Pasteurized milk feeding could improve weight gain and reduce sickness in calves [2, 3, 7]. For pasteurized milk, a longer holding time, instead of a higher temperature, was more effective in the inactivation of pathogenic organisms [8]. The acidification of milk has also been approved to be a labor-saving, simple and cost-effective method for calf feeding [6] and has been shown to prevent the rapid growth of pathogenic organisms in the digestive tract and reduce the incidence of infectious scours in calves 3 weeks of age and less [9, 10].

To our knowledge, considerable literature has been published concerning the difference between feeding pasteurized milk or acidified milk replacer [11–13], but there is a lack of published data on the difference between feeding pasteurized waste milk and acidified waste milk to calves. Furthermore, whole milk is generally considered to be the best feed for young calves. Therefore, this investigation was performed to elucidate the effects of feeding bunk tank milk (BTM), untreated waste milk (UWM), pasteurized waste milk (PWM) or acidified waste milk (AWM) on the growth performance, serum metabolism, immunity, and intestinal development in calves.

## Methods

### Animals, treatments and management

Eighty-four Holstein neonatal male calves with similar birth weight (43.6 ± 5.1 kg) were selected from the Modern Farming Feidong Farm (Hefei, Anhui, China). Each calf was fed 4.0 L colostrum immediately after birth and then assigned to one of four treatment groups with three calves/pen fed individually with seven replicates/treatment for each kind of milk. For each treatment, calves received the following different sources of milk from d 2 after birth for 21 d: bunk tank milk (BTM), untreated waste milk (UWM), pasteurized waste milk (PWM), and acidified waste milk (AWM). Bunk tank milk (BTM) was taken from the milking line twice daily. Untreated waste milk (UWM), which was composed of surplus colostrum, transitional milk, and milk from cows treated with veterinary drugs due to mastitis or other diseases, was collected twice daily into a specific tank. Pasteurized waste milk (PWM) was prepared by pasteurization of untreated waste milk at 72 °C for 15 s. Acidified waste milk (AWM) was acidified by the addition of 30 mL formic acid into 1 L of untreated waste milk, formic acid solution was diluted by 85% formic acid (Fucheng Chemical Reagent Factory, Tianjin, China) with water.

Calves received the milk at equal volumes twice daily via nipple buckets at 0700 h and 1700 h from d 2 in the amount of 3.0 L/meal, from d 8 in the amount of 3.5 L/meal, and from d 15 in the amount of 4.0 L/meal. The same pelleted calf starter was offered from d 4 until d 21. The nipple buckets were cleaned twice daily after milk feeding with a brush using hot tap water and a commercial detergent followed by rinsing with clear water. Calves were housed in pens with three calves as a replicate group on straw bedding and had free access to water during the experimental period. The experimental protocol was conducted in accordance with the practices outlined in the Guide for the Care and Use of Agriculture Animals in Agriculture Research and Teaching [14].

### Experimental sampling, measurements and chemical analysis

#### Milk composition

Milk samples were taken daily before feeding, preserved with potassium dichromate and stored at 4 °C until analyzed for milk fat, protein and solid non-fat (SNF) percentages using a near-infrared reflectance spectroscopy analyzer (Milk Scan 605, Foss Electric, Hillerød, Denmark) at the Beijing Dairy Cattle Center. The pH values were measured using a digital pH meter (PHB-4, Shanghai Hongyi instrument Limited, China). Milk acidity was determined by titration with NaOH, and phenolphthalein was used as the indicator. Total viable count (TVC) was calculated using the colony count on plates in the laboratory of Modern Farming Feidong Farm (Hefei, Anhui, China).

#### Growth performance measurement and diarrhea incidence

The feed intake of each pen was recorded every morning. The fact BW of all calves in the BTM, UWM, PWM and AWM groups were taken initially and on d 22 before feeding, and average daily feed intake (ADFI) and average daily gain (ADG) were calculated.

Fecal consistency scores for calves were determined daily based on a 1 to 5 system described as follows: 1 = normal, thick in consistency; 2 = normal, soft, less thick; 3 = abnormally thin but not watery; 4 = watery; and 5 = watery with abnormal coloring [15]. A fecal score of 2 and above was considered diarrhea, and gentamycin sulfate injections were administered to calves when fecal scores were 4 or higher.

#### Serum collection and analysis

Serum samples of all calves were taken on 48 h after born to detect TP concentration and assess passive immunity. On d 22 before morning feeding, 6 calves per treatment (BTM, UWM, PWM, AWM) were randomly chosen, and blood samples were collected via jugular venipuncture into Vacutainer tubes (Becton Dickinson, Franklin Lakes, NJ) before feeding. Samples were centrifuged at 3,000 × g for 15 min, and the supernatants (serum) were collected and frozen at −20 °C until analysis for the determination of total protein (TP), albumin (ALB), high density lipoprotein (HDL), low density lipoprotein (LDL), urea nitrogen (UN), creatinine (Cr), triglycerides (TG), total cholesterol (TC), bilirubin (BIL), glucagon (GC), growth hormone (GH), superoxide dismutase (SOD), malondialdehyde (MDA), glutathione peroxidase (GSH-px), immunoglobulin G (IgG), immunoglobulin A (IgA), immunoglobulin M (IgM), complement 3 (C3), complement 4 (C4), interleukin-1β (IL-1β), interleukin-6 (IL-6), interleukin-7 (IL-7), interleukin-8 (IL-8), interleukin-10 (IL-10), interleukin-22 (IL-22), and tumor necrosis factor-α (TNF-α).

The concentrations of TP, ALB, HDL, LDL, UN, Cr, TG, TC, BIL, SOD, MDA, GSH-px, IgA, IgM, C3, and C4 were determined using corresponding assay kits from Shandong BioBase Biotechnologies Inc. (Shandong, China) and Nanjing Jiancheng Bioengineering Institute (Jiangsu, China) in an automated biochemistry analyzer (Model GF-D200, Rainbow, Shandong, China). Aliquots of plasma serum were used to measure GC, GH and IgG using radioimmunoassay kits from HTA Co. Ltd. (Beijing, China) in an absorbance microplate reader (EXL 800, Bio-tek, Vermont, USA). The serum immune factors (IL-1β, IL-6, IL-7, IL-8, IL-10, IL-22, TNF-α) were analyzed using commercial ELISA kits from Shanghai HoraBio Inc. (Shanghai, China).

#### Small intestinal tissue sampling

On d 22 after morning feeding, the six calves selected for blood collection were harvested by exsanguination. The intestinal tracts of these calves were excised and divided into the following three segments: the duodenum, jejunum and ileum. The jejunal and ileal pH values were immediately measured in the mid-segment using a digital pH meter (PHB-4, Shanghai Hongyi instrument Limited, China). For gut histopathological and immunohistochemical analysis, approximately 2-cm lengths of the mid-jejunum and ileum were removed and flushed with ice-cold buffered PBS at pH 7.4 and immediately placed in a 4% formalin solution [16]. For mRNA analysis, jejunal mucosa and mesenteric lymph nodes were rinsed in saline and transferred to plastic vials, snap-frozen in liquid N, and stored at −80 °C until analysis [17].

#### mRNA abundance

Samples were selected for analysis of *IL-1β*, *IL-6*, *IL-8*, *IL-10*, *TNF-α*, and toll like receptor 4 (*TLR-4*). Total RNA was isolated using the method of Al-Trad et al. [18]. Total RNA was extracted from 100 mg of each sample using TRIzol reagent (Invitrogen, Carlsbad, CA) according to the manufacturer’s instructions. The quantity and quality of the isolated RNA were determined by absorbance at 260 and 280 nm. Total RNA (1 μg) was reverse transcribed using 1 μL Oligo-dT primer (Promega, Madison, WI, USA) and 0.625 μL RNasin (Promega, Madison, WI, USA) in a 25-μL reaction for 5 min at 72 °C and 60 min at 42 °C. The resulting first-strand cDNA was stored at −20 °C until used for real-time PCR. Semi-quantitative real-time PCR was carried out on an ABI7500 Real-time PCR (Applied Biosystems, Inc., Carlsbad, US) using GAPDH (forward: 5′-GTCTTCACTACCATGGAGAAGG-3′, reverse: 5′-TCATGGATGACCTTGGCCAG-3′) as an internal reference gene. Primers for quantitative rt-PCR were designed as follows: IL-1β: 5′-CCTCGGTTCCATGGGAGATG-3′ (forward) and 5′-AGGCACTGTTCCTCAGCTTC-3′ (reverse); IL-6: 5′-GCTGAATCTTCCAAAAATGGAGG-3′ (forward) and 5′-GCTTCAGGATCTGGATCAGTG-3′ (reverse); IL-8: 5′-ACACATTCCACACCTTTCCAC-3′ (forward) and 5′-ACCTTCTGCACCCACTTTTC-3′ (reverse); IL-10: 5′-CCTTGTCGGAAATGATCCAGTTTT-3′ (forward) and 5′-TCAGGCCCGTGGTTCTCA-3′ (reverse); TNF-α: 5′-TCCAGAAGTTGCTTGTGCCT-3′ (forward) and 5′-CAGAGGGCTGTTGATGGAGG- 3′ (reverse); and TLR-4: 5′-TATGAACCACTCCACTCGCTC-3′ (forward) and 5′-CATCATTTGCTCAGCTCCCAC-3′ (reverse). For each sample, the target gene and the control gene were run under duplex reaction conditions in duplicate. All reactions used the following protocol according to the instructions: 2 μL of sample cDNA, 10 μL 2× premix ExTaqTm II (Takara Bio, Inc., Kusatsu, Shiga, Japan), 0.8 μL of 10 μmol/L forward primers, 0.8 μL of 10 μmol/L reverse primers, 0.4 μL ROX correction dye, and the total volume was adjusted with nuclease-free H_2_O to 20 μL. For amplification, the following cycling conditions were used: 95 °C for 5 s, 60 °C for 30 s for 40 cycles after an initial denaturation step of 30 s at 95 °C, and then an elongation step at 70 °C for 15 s. A dissociation curve was achieved by melting DNA at 95 °C for 15 s, incubating at 60 °C for 1 min, ramping up to 95 °C for 30 s, and cooling down to 60 °C for 15 s. The relative gene expression was determined by quantitative RT-PCR and expressed using 2^-ΔΔCT^ methods as described by Livak and Schmittgen [19]; BTM was established as the control group. The relative expression of the target gene mRNA in each group was calculated as follows: ΔCT = CT (target gene) - CT (GAPDH), and ΔΔCT = ΔCT (treated group) - ΔCT (BTM group).

#### Histopathological scoring

The intestinal segments fixed with formalin solution were prepared using paraffin embedding, and 4-μm thickness sections were cut and then stained with hematoxylin-eosin (HE) for microscopic examination under 10×, 20×, and 60× magnifications using an Olympus BX41 microscope (Olympus, Tokyo, Japan) equipped with a Canon EOS 550D camera head (Canon, Tokyo, Japan) [20]. The bunk tank milk group was established as the control group.

Inflammation was scored using four aspects, including epithelial necrosis (0 = none; 1 = mild, <10%; 2 = moderate, 11-20%; 3 = severe, ulceration), leukocyte infiltration (0 = normal, <10%; 1 = mild, 11-15%; 2 = moderate, 16-20%; 3 = severe, >20%), central lacteal expansion (0 = none; 1 = mild; 2 = moderate; 3 = severe), and submucosal edema (0 = none; 1 = mild; 2 = moderate; 3 = severe). The sum of the four parameter scores represents the overall inflammation status of each intestinal segment, which were evaluated as follows: 0 = normal, 1–5 = mild, 6–10 = moderate, and 11–12 = severe.

#### Immunohistochemical scoring

After deparaffinization and hydration using xylenes, slides were subjected to a sodium citrate buffer solution for antigen retrieval. Endogenous peroxidase activity was quenched with 3% H_2_O_2_ in methanol for 20 min, after which slides were incubated with goat serum (Zhongshan Golden Bridge Biotechnology Co., Beijing, China) of the species for 15 min.

Next, sections were incubated for 1 h at 37 °C with a mouse monoclonal TLR-4 antibody (76B357.1, Abcam, England, United Kingdom). Afterwards, slides were incubated with a biotinylated secondary antibody for 30 min at 37 °C. Bound antibody conjugates were visualized using 3,3′-DAB (Zhongshan Golden Bridge Biotechnology Co., Beijing, China) as a chromogen. The slides were counterstained with hematoxylin for microscopic examination under a 40× magnification using an Olympus BX41 microscope (Olympus, Tokyo, Japan) equipped with a Canon EOS 550D camera head (Canon, Tokyo, Japan).

Immunohistochemical scores were evaluated using two aspects, including the percentage of immunoreactive cells (0 = 0–1%; 1 = 1–10%; 2 = 10–50%; 3 = 50–80%; 4 = 80–100%) and staining intensity (0 = negative; 1 = weak; 2 = moderate; 3 = strong), according to Soslow et al. [21]. The multiplied score of the two parameters represents the overall immunoreactivity of each intestinal segment, which was evaluated as follows: 0 = negative, 1–4 = weak, 5–8 = moderate, and 9–12 = strong.

### Statistical analysis

Data were analyzed using the GLM procedure in SAS 9.0. Values are expressed as the means ± SE. The model for treatment differences was:

$$ {\mathrm{Y}}_{\mathrm{i}\mathrm{j}}=\upmu + {\mathrm{T}}_{\mathrm{i}} + {\mathrm{C}}_{\mathrm{j}} + {\mathrm{e}}_{\mathrm{i}\mathrm{j}} $$

where

μ = overall mean;

T_i_ = the effect of treatment (i = 1 to 4);

C_j_ = the effect of calf (j = 1 to 21 of body weight and fecal score; j = 1 to 7 of feed intake; j = 1 to 6 of serum and intestinal index);

e_ij_ = random error.

Significant differences in mean values were evaluated using Duncan’s multiple range test. A significance level of *P* < 0.05 was used.

## Results

### Milk composition

Milk composition of BTM, UWM, PWM and AWM is shown in Table 1. There were no significant differences (*P* > 0.05) in milk protein among the four kinds of milk. Milk fat and SNF percentage in UWM were significantly higher than BTM, PWM, and AWM. The pH value in AWM decreased and acidity increased with the addition of formic acid. TVC in AWM was higher than PWM.

**Table 1:** Milk composition of different sources of milk

Items	Treatments^c^			
	BTM	UWM	PWM	AWM
Milk protein, %	3.26 ± 0.04	3.54 ± 0.08	3.62 ± 0.05	3.35 ± 0.16
Milk fat, %	4.22 ± 0.24^b^	6.30 ± 0.44^a^	4.50 ± 0.07^b^	4.33 ± 0.10^b^
SNF^﻿d﻿^	13.0 ± 0.04^b^	15.1 ± 0.08^a^	13.3 ± 0.05^b^	13.4 ± 0.20^b^
pH	6.71 ± 0.02^a^	6.63 ± 0.05^a^	6.58 ± 0.02^a^	4.65 ± 0.06^b^
Acidity	12.7 ± 0.18^b^	14.3 ± 0.65^b^	15.4 ± 0.33^b^	72.9 ± 1.96^a^
TVC﻿^d﻿^, × 10^3^	91.0 ± 51.4^ab^	87.8 ± 49.6^ab^	0.18 ± 0.15^b^	520 ± 300^a^

^a,b^Means within a row not sharing a common superscript letter are significantly different (*P* < 0.05)

^c^ *BTM* bunk tank milk group, *UWM* untreated waste milk group, *PWM* pasteurized waste milk group, *AWM* acidified waste milk group

^d^ *SNF* solid non-fat, *TVC* total viable count

### Growth performance and health status

All calves in three groups had similar BW at birth (Table 2). Body weight for calves in the UWM group was higher than the BTM group, and weight gain in the UWM group was higher than the BTM, PWM, and AWM groups. ADG in the BTM group was lower than the UWM, PWM and AWM groups. Calves in the BTM and AWM groups consumed more starter than the UWM group. The Fecal score in the AWM group was significantly lower than the BTM, UWM, and PWM groups. Twelve calves experienced diarrhea in the BTM and PWM groups, while 14 or 11 calves had diarrhea in the UWM or AWM groups, respectively.

**Table 2:** Growth performance and health status of calves feeding on different sources of milk

Items^d^	Treatments^c^			
	BTM	UWM	PWM	AWM
Initial body weight, kg	43.7 ± 1.13	43.4 ± 1.28	43.1 ± 0.87	44.4 ± 0.91
Body weight, kg	49.3 ± 1.26^b^	54.4 ± 1.85^a^	52.5 ± 1.36^ab^	52.5 ± 1.37^ab^
Weight gain, kg	5.43 ± 0.85^b^	11.0 ± 1.09^a^	9.53 ± 0.92^b^	8.56 ± 0.95^b^
ADG, g/d	258 ± 40.4^b^	525 ± 51.9^a^	454 ± 43.9^a^	408 ± 45.2^a^
ADFI, g/d	34.4 ± 6.64^a^	21.3 ± 3.89^b^	29.4 ± 4.72^ab^	36.2 ± 4.33^a^
Fecal score	2.99 ± 0.07^a^	2.94 ± 0.06^a^	2.94 ± 0.10^a^	2.72 ± 0.05^b^
Diarrhea, no. of animals	12	14	12	11

^a,b^Means within a row not sharing a common superscript letter are significantly different (*P* < 0.05)

^c^ *BTM* bunk tank milk group, *UWM* untreated waste milk group, *PWM* pasteurized waste milk group, *AWM* acidified waste milk group

^d^ *ADG* average daily gain, *ADFI* average daily feed intake

### Serum metabolites and growth index

Serum TP content were similar (*P* ≥ 0.05) in the four experimental groups (Table 3) at 48 h after feeding. There were no significant differences (*P* > 0.05) among the four treatments for LDL, UN, Cr, and BIL. Serum TP, ALB, TC, and GC concentrations were significantly higher in the UWM group than the BTM, PWM, and AWM groups. Untreated waste milk feeding calves expressed higher HDL, TG and GH concentrations, the BTM group expressed lower HDL and GH, and the AWM group expressed lower TG.

**Table 3:** Serum metabolites and growth index of calves feeding on different sources of milk

Items^d^	Treatments^c^			
	BTM	UWM	PWM	AWM
TP on 48 h, g/L	6.02 ± 0.14	5.98 ± 0.13	5.55 ± 0.14	5.61 ± 0.13
TP, g/L	56.2 ± 5.16^b^	71.3 ± 2.46^a^	48.5 ± 2.91^b^	45. 7 ± 4.70^b^
ALB, g/L	16.3 ± 2.01^b^	22.5 ± 1.91^a^	13.8 ± 0.60^b^	13.0 ± 1.39^b^
HDL, mmol/L	1.03 ± 0.16^b^	1.62 ± 0.28^a^	1.12 ± 0.13^ab^	1.11 ± 0.08^ab^
LDL, mmol/L	0.32 ± 0.18	0.47 ± 0.11	0.29 ± 0.08	0.19 ± 0.04
UN, mmol/L	2.86 ± 0.23	3.17 ± 0.16	2.83 ± 0.29	2.49 ± 0.28
Cr, μmol/L	79.0 ± 8.07	67.3 ± 3.86	63.8 ± 3.28	71.8 ± 8.62
TG, mmol/L	0.30 ± 0.05^ab^	0.36 ± 0.05^a^	0.26 ± 0.03^ab^	0.18 ± 0.02^b^
TC, mmol/L	1.48 ± 0.25^b^	2.25 ± 0.33^a^	1.52 ± 0.18^b^	1.38 ± 0.09^b^
BIL, μmol/L	6.90 ± 0.77	6.93 ± 0.71	4.78 ± 0.43	6.12 ± 1.32
GC, pg/mL	559 ± 84.6^b^	811 ± 21.4^a^	320 ± 76.2^b^	203 ± 27.0^b^
GH, ng/mL	1.26 ± 0.27^b^	2.00 ± 0.18^a^	1.58 ± 0.09^ab^	1.82 ± 1.15^ab^

^a,b^Means within a row not sharing a common superscript letter are significantly different (*P* < 0.05)

^c^ *BTM* bunk tank milk group, *UWM* untreated waste milk group, *PWM* pasteurized waste milk group, *AWM* acidified waste milk group

^d^ *TP* total protein, *ALB* albumin, *HDL* high density lipoprotein, *LDL* low density lipoprotein, *UN* urea nitrogen, *Cr* creatinine, *TG* triglyceride, *TC* total cholesterol, *BIL* bilirubin, *GC* glucagon, *GH* growth hormone

### Immune and antioxidant performance

There were no significant differences (*P* > 0.05) in SOD, C3, C4, IL-6, IL-7, IL-8, IL-10, and IL-22 concentrations among the four treatments (Table 4) before feeding on d 22. The MDA concentration was the highest in the PWM group and higher in the AWM group compared to the BTM and UWM groups. Compared with the other three groups, calves expressed higher GSH-px and IgG concentrations in the BTM group and IgA in the UWM group. Serum IgM concentration was higher in the UWM group than the AWM group, and IL-1β and TNF-α were higher in the UWM and PWM groups compared to the BTM and AWM groups (Table 5).

**Table 4:** Antioxidant and immunestatus of calves feeding on different sources of milk

Items^d^	Treatments^c^			
	BTM	UWM	PWM	AWM
SOD, U/mL	56.5 ± 0.51	54.1 ± 2.03	57.2 ± 0.88	57.9 ± 0.84
MDA, nmol/L	1.28 ± 0.22^c^	1.37 ± 0.29^c^	1.91 ± 0.09^a^	1.58 ± 0.22^b^
GSH-px, μmol/mL	79.1 ± 33.2^a^	44.5 ± 23.8^b^	51.7 ± 27.0^b^	43.1 ± 22.6^b^
IgG, pg/mL	0.87 ± 0.28^a^	0.58 ± 0.08^b^	0.57 ± 0.10^b^	0.43 ± 0.13^b^
IgA, g/L	0.66 ± 0.03^b^	0.82 ± 0.04^a^	0.71 ± 0.05^b^	0.69 ± 0.03^b^
IgM, g/L	2.49 ± 0.17^ab^	2.89 ± 0.20^a^	2.53 ± 0.18^ab^	2.17 ± 0.05^b^
C3, mg/dL	0.20 ± 0.04	0.31 ± 0.05	0.25 ± 0.04	0.22 ± 0.05
C4, mg/dL	2.23 ± 0.31	2.11 ± 0.23	2.26 ± 0.26	2.54 ± 0.23

^a,b^Means within a row not sharing a common superscript letter are significantly different (*P* < 0.05)

^c^ *BTM* bunk tank milk group, *UWM* untreated waste milk group, *PWM* pasteurized waste milk group, *AWM* acidified waste milk group

^d^ *SOD* superoxide dismutase, *MDA* malondialdehyde, *GSH-px* glutathione peroxidase, *IgG* immunoglobulin G, *IgA* immunoglobulin A, *IgM* immunoglobulin M, *C3* complement 3, *C4* complement 4

**Table 5:** Serum immune factors of calves feeding on different sources of milk

Items^d^	Treatments^c^			
	BTM	UWM	PWM	AWM
IL-1β, ng/L	60.0 ± 5.49^b^	70.3 ± 7.98^a^	72.3 ± 3.04^a^	61.7 ± 4.14^b^
IL-6, ng/L	610 ± 12.8	583 ± 26.6	599 ± 49.4	602 ± 36.0
IL-7, ng/L	24.9 ± 1.27	26.6 ± 1.22	26.3 ± 1.18	24.8 ± 1.28
IL-8, ng/L	86.9 ± 2.80	83.8 ± 3.44	91.6 ± 4.26	88.9 ± 2.38
IL-10, ng/L	264 ± 21.5	269 ± 26.8	277 ± 20.1	283 ± 13.9
IL-22, ng/L	0.49 ± 0.02	0.49 ± 0.05	0.51 ± 0.01	0.54 ± 0.02
TNF-α, ng/L	258 ± 20.7^b^	299 ± 27.2^a^	301 ± 13.8^a^	273 ± 6.07^b^

^a,b^Means within a row not sharing a common superscript letter are significantly different (*P* < 0.05)

^c^ *BTM* bunk tank milk group, *UWM* untreated waste milk group, *PWM* pasteurized waste milk group, *AWM* acidified waste milk group

^d^ *IL-1β* interleukin-1β, *IL-6* interleukin-6, *IL-7* interleukin-7, *IL-8* interleukin-8, *IL-10* interleukin-10, *IL-22* interleukin-22, *TNF-α* tumor necrosis factor-α

### mRNA expression

There were no differences (*P* > 0.05) in mRNA expression of jejunal *IL-6* and *TNF-α*, and the *TNF-α* and *TLR-4* in the mesenteric lymph nodes (Table 6). Compared with the other three groups, calves feeding on UWM increased jejunal *IL-10* expression, and feeding on AWM increased *IL-8* mRNA expression in the mesenteric lymph nodes.

**Table 6:** MRNA expression of immune factors on jejunal mucosa and mesenteric lymph nodes of calves

Items^d^	Treatments^c^			
	BTM	UWM	PWM	AWM
Jejunal mucosa				
*IL-1β*	1.00 ± 0.22^b^	6.32 ± 0.92^a^	1.67 ± 0.67^ab^	3.00 ± 0.90^ab^
*IL-6*	1.00 ± 0.27	4.38 ± 0.15	1.86 ± 0.46	3.76 ± 1.30
*IL-8*	1.00 ± 0.63^b^	6.11 ± 0.87^a^	1.80 ± 0.47^b^	4.41 ± 1.18^a^
*IL-10*	1.00 ± 0.64^b^	10.49 ± 0.40^a^	1.41 ± 0.72^b^	3.06 ± 0.83^b^
*TNF-α*	1.00 ± 0.08	4.88 ± 0.45	0.98 ± 0.05	1.45 ± 0.26
*TLR-4*	1.00 ± 0.16^b^	0.88 ± 0.06^b^	7.31 ± 0.67^ab^	10.09 ± 1.82^a^
Mesenteric lymph nodes				
*IL-1β*	1.00 ± 0.23^b^	1.73 ± 0.24^ab^	1.73 ± 0.26^ab^	3.30 ± 0.40^a^
*IL-6*	1.00 ± 0.13^b^	1.13 ± 0.37^ab^	1.03 ± 0.30^ab^	1.95 ± 0.28^a^
*IL-8*	1.00 ± 0.70^b^	1.33 ± 0.58^b^	0.72 ± 0.21^b^	6.30 ± 0.83^a^
*IL-10*	1.00 ± 0.33^b^	1.55 ± 0.16^ab^	1.35 ± 0.18^b^	2.22 ± 0.08^a^
*TNF-α*	1.00 ± 0.12	0.98 ± 0.30	0.94 ± 0.1	0.56 ± 0.24
*TLR-4*	1.00 ± 0.09	1.36 ± 0.26	0.82 ± 0.54	1.60 ± 0.47

^a,b^Means within a row not sharing a common superscript letter are significantly different (*P* < 0.05)

^c^ *BTM*, bunk tank milk group, *UWM* untreated waste milk group, *PWM* pasteurized waste milk group, *AWM* acidified waste milk group

^d^ *IL-1β* interleukin-1β, *IL-6* interleukin-6, *IL-8* interleukin-8, *IL-10* interleukin-10, *TNF-α* tumor necrosis factor-α, *TLR-4* toll-like receptor-4

For jejunum, *IL-1β* mRNA expression was higher in the UWM group than the BTM group, *IL-8* mRNA expression was improved in the UWM and AWM groups than the BTM group, and *TLR-4* mRNA expression was higher in the AWM group than the UWM and BTM groups. In the mesenteric lymph nodes, mRNA expression of *IL-1β* and *IL-6* was higher in the AWM group than the BTM group, and *IL-10* was higher in the AWM group than the BTM and PWM groups.

### Intestinal development

**Fig. 1:**
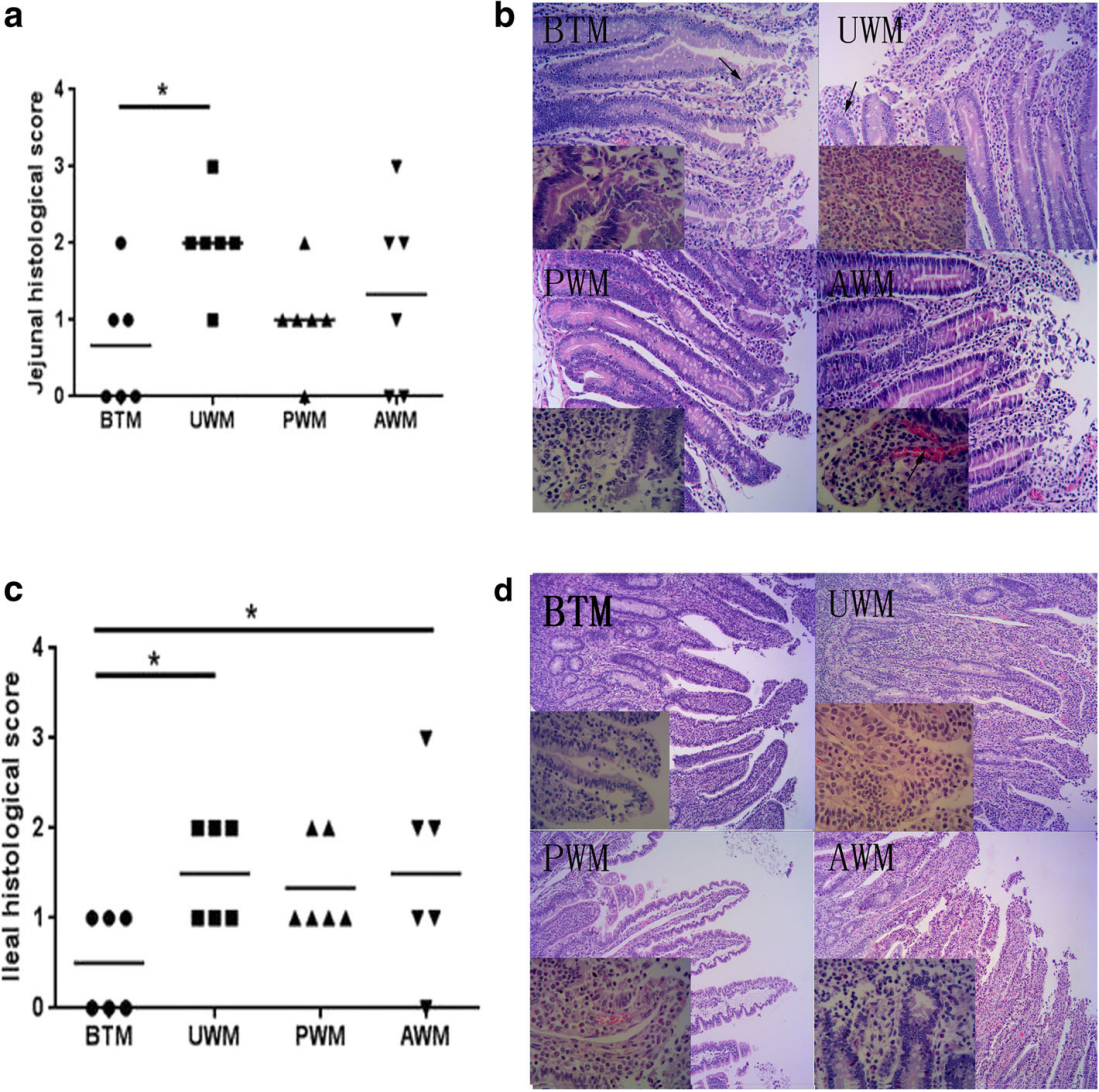
Histological scores and light micrographs of hematoxylin and eosin stained jejunal and ileal sections of calves feeding on different sources of milk. **a** Jejunal histological scores. **b** Representative photomicrographs of hematoxylin and eosin stained jejunal sections in the BTM, UWM, PWM, and AWM groups (H & E 20× and 60× original magnification). **c** Ileal histological scores. **d** Representative photomicrographs of hematoxylin and eosin stained ileal sections in the BTM, UWM, PWM, and AWM groups (H & E 10× and 60× original magnification). Intestine pictures from calves receiving bunk tank milk, untreated waste milk, pasteurized waste milk, or acidified waste milk after feeding on colostrum, respectively. The jejunal and ileal basal structure of the calves in the four treatments was clear and intact. Slight epithelial lesions (arrows) were observed in the jejunum and ileum of the BTM and UWM fed calves, and leukocyte infiltration (arrows) was found in the UWM and AWM fed calves

Jejunal and ileal pH values were similar (*P* > 0.05) among the four experimental groups. The jejunum of the calves in the four treatments were well developed with intact structure, with long, intensive and uniform villi and deep crypts, with a few tissue abscesses in the villus apex (Fig. 1b). The ileal basal structure of the calves in the four treatments was clear and intact, but villi of the ileum from calves in the BTM group were less uniform, where villi of the ileum in the UWM, PWM and AWM groups were more uniform with some apical abscesses (Fig. 1d). The jejunum and ileum in all four experimental treatments expressed mild submucosa edema and no central lacteal expansion (Fig. 1a, c). Slight epithelial lesions were observed in the jejunum and ileum of the BTM and UWM calves, and leukocyte infiltration was found in the UWM and AWM calves. Numerically, the inflammation scores of the jejunum increased in the UWM and AWM groups compared to the BTM group and increased in the ileum in the AWM group compared to the BTM group (Table 7).

**Table 7:** Intestinal development of calves feeding on different sources of milk

Items^d^	Treatments^c^			
	BTM	UWM	PWM	AWM
pH				
Jejunum	6.05 ± 0.07	5.94 ± 0.12	5.95 ± 0.17	6.13 ± 0.10
Ileum	6.68 ± 0.10	6.53 ± 0.32	6.44 ± 0.47	6.84 ± 0.14
Inflammation scores				
Jejunum	0.67 ± 0.67^b^	2.33 ± 0.33^a^	1.33 ± 0.33^ab^	2.33 ± 0.33^a^
Ileum	0.67 ± 0.33^b^	1.33 ± 0.33^ab^	1.00 ± 0.00^ab^	2.00 ± 0.58^a^
IHS				
Jejunum	1.33 ± 0.33^b^	2.67 ± 0.67^ab^	4.00 ± 1.15^a^	4.00 ± 0.00^a^
Ileum	1.67 ± 0.33^b^	4.00 ± 0.00^a^	4.67 ± 0.67^a^	5.33 ± 0.67^a^

^a,b^Means within a row not sharing a common superscript letter are significantly different (*P* < 0.05)

^c^ *BTM* bunk tank milk group, *UWM* untreated waste milk group, *PWM* pasteurized waste milk group, *AWM* acidified waste milk group

^d^ *IHS* immunohistochemical scores

**Fig. 2:**
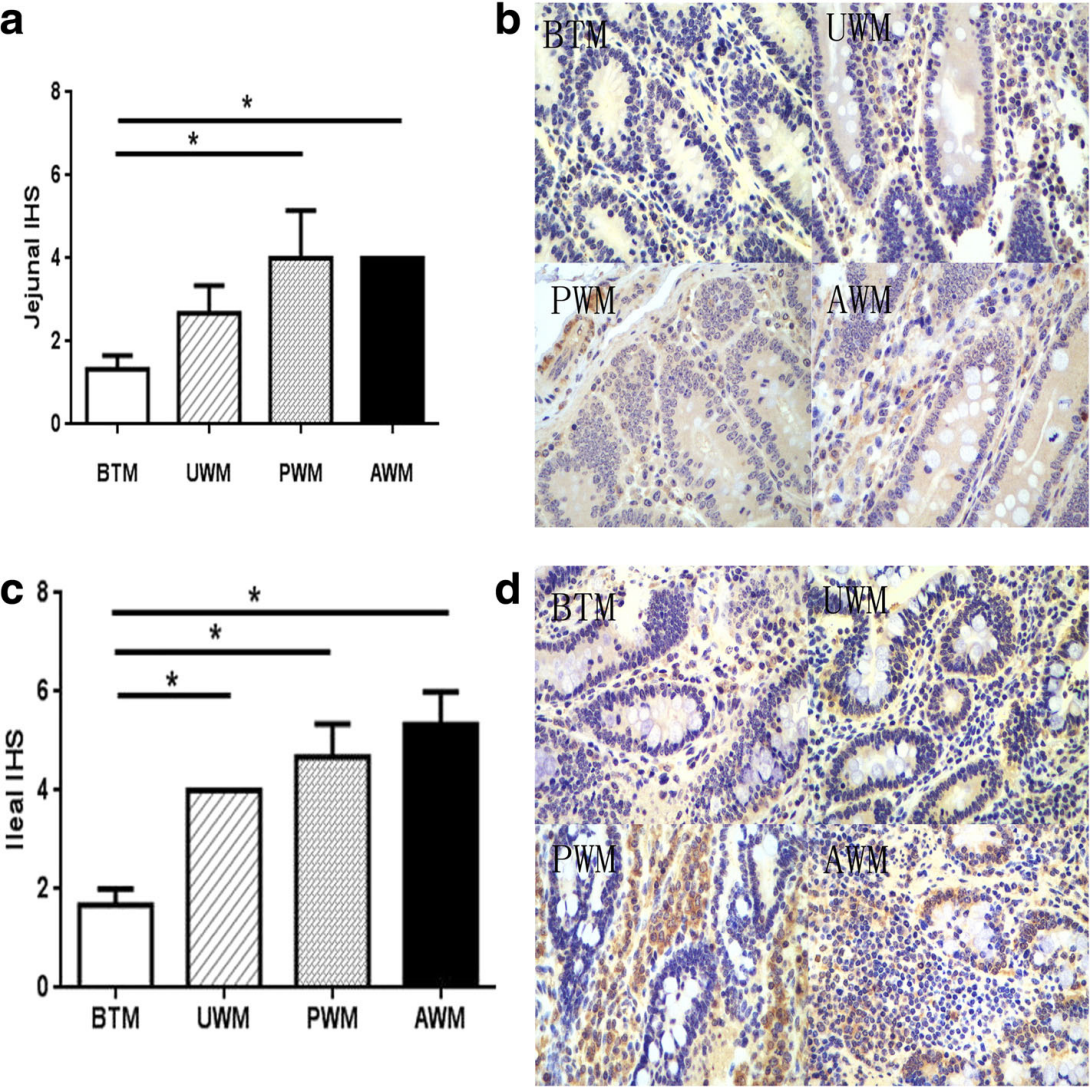
Immunohistochemical scores and light micrographs of DAB stained jejunal and ileal sections of calves. **a** Jejunal immunohistochemical scores. **b** Representative photomicrographs of DAB stained jejunal sections in the BTM, UWM, PWM, and AWM groups (H & E 40 × original magnification). **c** Ileal immunohistochemical scores. **d** Representative photomicrographs of DAB stained ileal sections in the BTM, UWM, PWM, and AWM groups (H & E 40 × original magnification). Intestine pictures from the calves receiving bunk tank milk, untreated waste milk, pasteurized waste milk, or acidified waste milk after feeding colostrum, respectively. Analyses of the jejunum and ileum sections from all the calves revealed that TLR-4 cells exhibited brown particles in the epithelium, which was clearly detected in the BTM group, a few detected in the UWM group, and more were observed in the PWM and AWM groups

Immunohistochemical analysis was used to localize TLR-4 cells in the jejunum and ileum of calves subjected to different kinds of milk. The analyses of jejunum and ileum sections from all calves revealed that TLR-4 cells exhibited brown particles in the epithelium, which were clearly detected in the BTM group, a few existed in the UWM group, and more were observed in the PWM and AWM groups (Fig. 2b, d). The jejunal IHS was higher in the PWM and AWM groups than the BTM group. Compared with the other three groups, calves feeding on BTM had lower ileal IHS (Fig. 2a, c).

## Discussion

### Growth and health

On d 22, calves fed untreated waste milk had gained more body weight than those fed bunk tank milk, but more calves experienced diarrhea. We speculated the weight gain due to the high amount of milk fat and SNF percentages in the UWM, which contained excessive colostrum and transitional milk from the farm [22–24]. An experiment conducted on calves from d 3 to d 56 concluded that weight gain and health parameters were not influenced by feeding on untreated waste milk, pasteurized waste milk, or bulk milk [1]. Similar results have also been reported in the literature [7, 25–27], which did not observe detrimental effects of waste milk on growth performance and calf health.

The advantages of feeding pasteurized waste milk have been observed in previous studies with 300 or 438 calves [2, 3], which demonstrated higher mean weight gain and lower diarrhea incidence. Similarly, growth performance and calf health were comparable between the BTM and PWM groups in this study, except for a higher ADG in the PWM group.

The acidification of milk replacer for use in rearing calves has been studied by several investigators [28–30], and the digestibility of dietary nutrients and growth performance could be improved when the pH was decreased to a proper range [11]. The most apparent difference was the decrease in pH in waste milk and the fecal score of calves fed with AWM compared to the increase in ADFI and ADG, which may be because the addition of formic acid enhanced dietary flavor thus promoting animal appetite [31].

### Serum metabolites and growth index

When the serum TP level is lower than 5.2 g/dL, 24–48 h after birth then it is referred to as failure of passive transfer [32]. All calves in the present study achieved passive transfer. The changes in serum TP, ALB, and BUN reflects the utilization efficiency of protein [33] and changes in TG and TC reflects lipid metabolism [34, 35]. Serum total protein can be inhibited when dietary nutrients are imbalanced or when the feed intake is decreased [34, 36]. The untreated waste milk fed calves had higher serum TP, ALB, HDL, TG and TC than the BTM fed calves. This mirrored milk protein content and ADFI demonstrates that a highly nutritional milk could enhance protein and lipid synthesis ability [37]. The untreated waste milk had more of a beneficial effect than the BTM in GH and GC, which was consistent with weight gain and average daily gain.

### Serum antioxidant and immune performance

The body antioxidant system, which prevents the toxic effects of free oxygen and its metabolites, is normally under a dynamic equilibrium between the generation and removal of free oxidative radicals and is highly related to animal health. Calves could acquire an antioxidative defense ability from milk after birth. Superoxide dismutase, which is the main parameter to assess oxidative status [38], catalyzes the dismutation of the superoxide radical anion [39]. Bovine milk contains Cu-Zn superoxide dismutase (Cu/Zn-SOD) [40, 41]. In the present study, serum SOD concentration in calves was not influenced by different sources of milk. The serum MDA level is used to monitor the extent of lipid peroxidation by reactive oxygen species [42] and reflects the degree of damage directly caused by free radicals. Calves feeding on PWM and AWM exhibited a high degree of destruction by free radicals based on high serum MDA level, especially in the pasteurized waste milk feeding group. Superoxide can be catalyzed and converted into water by GSH-px [43]. Glutathione peroxidase concentration in calves fed BTM was much higher compared to those fed UWM, PWM and AWM. We postulate that when the antioxidant mechanisms of SOD and GSH-px were activated by bunk tank milk, the oxidation by MDA production was lowered. This finding indicates that a high quality nutrient content and microbial activity in bunk tank milk is helpful in establishing antioxidative defense mechanisms in calves.

Complement 3 and C4 are the intrinsic composition of the complement system. The immune system will function properly if C3 and C4 content is maintained within a suitable range, otherwise, immunity defense mechanisms can be reduced. Complement 3 and C4 concentrations of the four groups did not vary in this study, reflecting a stable immunity for all of the calves.

The regulation mechanism of humoral immunity can be directly reflected by serum immune-globulins levels. Researchers have documented that the serum IgG content of calves with diarrhea was clearly lower than healthy calves and that there is a positive relationship between serum IgG concentration and diarrhea incidence of calves [44]. Additionally, Tu [11] noted serum IgA and IgM was highest when milk pH replacer reached 5.0. However, higher IgG was observed in calves receiving BTM, and higher IgA and IgM was observed in calves receiving UWM, and no positive correlation was observed between IgG content and calf diarrhea.

### Serum immune factors and mRNA expression

Interleukins are a class of immune factors that play a role in regulating inflammation and immune response initiated by infection and injury [45]. Therefore, interleukins, such as IL-6, IL-8 and IL-10, are indicators of inflammation [46, 47]. The tumor necrosis factor participates in cell mediated immune response [48] and plays an important role in resisting and defending against intracellular viruses and mycoplasma [49, 50]. Toll-like receptor-4 recognizes microbial and inflammatory responses.

Studies that observed the effects of strains on the serum and mucosal immune factors varied, where *E. coli* caused diarrhea in piglets and increased the serum TNF-α, lipopolysaccharide stimulated IL-1, IL-6, IL-8 and IL-12 [51], but *Lactobacillus* improved IL-6 and IL-10 [52]. In the present study, calves feeding on UWM, PWM or AWM exhibited some up-regulation of immune factors in the serum, jejunal mucosa and mesenteric lymph nodes, which may be the results of different species and quantities of microbial activity, such as serum IL-1β and TNF-α of UWM and PWM feeding groups, four immune-factor of jejunal mucosa in UWM or AWM groups, and IL-1β, IL-6, IL-8, IL-10 in mesenteric lymph nodes of AWM feeding group.

### Intestinal development

Woodford [28] indicated that an acidified diet may decrease the pH only in the abomasum and that it would be neutralized by the pancreatic juice when the digesta reached the small intestine. The different sources of milk did not change the jejunal and ileal pH in calves, which agrees with the findings of a previous study on acidified milk replacer [11].

Normally, there is a dynamic balance between the intestinal microbial activity and the host, and calves can experience a series of diseases when this balance was destroyed [53]. Injury to the mucosa could damage the intestinal epithelial barrier function, which then can induce enteritis and diarrhea [54]. In the present study, slight apical abscissions, epithelial lesions, leukocyte infiltration and submucosal edemas were observed in all four milk feeding groups. In an experiment conducted on acidified milk replacer, Tu [11] noted the intestinal architecture can be well preserved when liquid pH equals 5.5 or 5.0. However, it appears from the histological scores that the acidified waste milk and untreated waste milk caused inflammation of the jejunum and ileum, and the intestine of the calves in the bunk tank milk feeding group were relatively healthier.

Immunohistochemical analyses of the jejunal and ileal tissues revealed that TLR-4 cells were primarily localized in the lamina propria and scattered in the epithelium [55]. Correspondingly, waste milk feeding increased the percentage of TLR-4 cells in the jejunum and ileum, which indicated an inflammatory response [55].

## Conclusions

Growth performance was not improved by bunk tank milk feeding as we expected, and untreated waste milk demonstrated the highest weight gain and average daily gain in the calves. Additionally, the acidified waste milk promoted daily feed intake. However, calves in the bunk tank milk feeding group had better antioxidant capacity than that in the pasteurized waste milk group. In addition, the small intestine of calves receiving bunk tank milk was healthier. From a nutritional and health point of view, bunk tank milk is the best choice for calf raising. All calves feeding on waste milk experienced varying degrees of enteritis. The efficiency of feeding pasteurized and acidified waste milk are comparable, and the acidification of waste milk is an acceptable labor-saving and diarrhea preventing feed for young calves.

## Abbreviations

ADFI: Average daily feed intake
ADG: Average daily gain
ALB: Albumin
AWM: Acidified waste milk
BIL: Bilirubin
BTM: Bunk tank milk
C3: Complement 3
C4: Complement 4
Cr: Creatinine
Cu/Zn-SOD: Cu-Zn superoxide dismutase
GC: Glucagon
GH: Growth hormone
GSH-px: Glutathione peroxidase
HDL: High density lipoprotein
HE: Hematoxylin-eosin
IgA: Immunoglobulin A
IgG: Immunoglobulin G
IgM: Immunoglobulin M
IHS: Immunohistochemical scores
IL-10: Interleukin-10
IL-1β: Interleukin-1β
IL-22: Interleukin-22
IL-6: Interleukin-6
IL-7: Interleukin-7
IL-8: Interleukin-8
LDL: Low density lipoprotein
MDA: Malondialdehyde
PWM: Pasteurized waste milk
SNF: Solid non-fat
SOD: Superoxide dismutase
TC: Total cholesterol
TG: Triglycerides
TLR-4: Toll like receptor-4
TNF-α: Tumor necrosis factor-α
TP: Total protein
TVC: Total viable count
UN: Urea nitrogen
UWM: Untreated waste milk
